# Diagnosis and treatment of multiple myeloma and AL amyloidosis with focus on improvement of renal lesion

**DOI:** 10.1007/s10157-012-0684-5

**Published:** 2012-09-13

**Authors:** Kenshi Suzuki

**Affiliations:** Department of Hematology, Japanese Red Cross Medical Center, 4-1-22 Hiroo, Shibuya-ku, Tokyo, 150-8935 Japan

**Keywords:** Multiple myeloma, AL amyloidosis, ASCT, Renal insufficiency

## Abstract

Multiple myeloma (MM) and AL amyloidosis are caused by the expansion of monoclonal plasma cells and secretion of dysproteinemia (Bence Jones protein and free light chain) and some patients require the hemodialysis. Myeloma kidney is mainly caused by the cast nephropathy of the distal tubuli, whereas, AL amyloid-protein is mainly deposited in glomeruli with massive fibrillar involvement. Therefore, almost MM patients presents a symptom of renal insufficiency, whereas, almost patients of AL amyloidosis present a nephrotic syndrome with severe hypoalbuminemia. These two diseases have some similar characteristics such as up-regulation of cyclin D1 gene by 11:14 chromosomal translocation. High-dose chemotherapy supported with autologous peripheral blood stem cells is effective for these two diseases. However, they are still difficult to be cured and require long-term disease control. In recent years, introduction of novel agents has changed their treatment strategies from the palliation therapy to the clinical cure.

## Introduction

Multiple myeloma (MM) is an incurable disease with high incidence rate in the elderly. Responsiveness to treatments varies largely among the patients due to high heterogeneity of MM. Decision of the treatment has been a difficult issue in MM. However, changes can be seen in its treatment strategies since good quality of response can be realistically obtained due to an introduction of novel drugs (bortezomib, lenalidomide, and thalidomide). This article reviews the latest trend and the future perspective of treatment for MM which has advanced remarkably in recent years.

MM and AL amyloidosis are similar diseases resulting from clonal proliferation and dysfunction of plasma cells, in which renal dysfunction due to deposition of immunoglobulin (M protein/amyloid) or other causes are frequently observed. Since exacerbation of renal function is closely associated with the prognosis of patients, maintenance or improvement of renal function by managing the underlying disease is required. In recent years, stratification of myeloma as high-risk and standard-risk by Mayo group has been introduced [1]. Deletion of 17p by FISH, t (14:16), Cytogenetic hypodiploidy, and β2-microglobulin >5.5 and LDH level >upper limit of normal are high risk sign. T (4:14) and cytogenetic deletion 13 are considered as intermediate risk by the reasons of overcoming with new drugs. After that, IMWG stratification is also published; Standard-risk were Hyperdiploidy (45 % of MM mainly IgG type and aged patients), t(11;14)(q13;q32) CCND1↑, and t(6;14) CCND3↑. Intermediate-risk were t(4;14)(p16;q32) MMSET↑ and deletion 13 or hypodiploidy by conventional karyotyping. High-risk were 17p deletion, t(14;16)(q32;q23) C-MAF↑, and t(14;20)(q32;q11) MAFB↑.

We classified AL amyloidosis into four groups as follows; cardiac, renal, gastrointestinal and pulmonary amyloidosis, and the others according to the main organ with AL amyloid materials deposition. In this decade, novel agents (bortezomib, thalidomide and lenalidomide) have become available to treat multiple myeloma in Japan. In this article, we review the recent trend for the diagnosis and treatment strategies of multiple myeloma and AL amyloidosis by focusing on how to improve renal lesion.

## Diagnosis and treatment of multiple myeloma

### Historical perspective

In 1962, Bergsagel et al. [2] reported that l-phenylalanine mustard (melphalan) could induce remissions in approximately one third of patients with MM. In 1967, Salmon et al. [3] reported that high doses of glucocorticoids could induce remissions in patients with refractory or relapsing MM. Combination therapy with melphalan and prednisolone in 1969 by Alexanian et al. [4] showed a better result than melphalan alone.

However, the response rate with alkylators and corticosteroids was only approximately 50 %, and CR was rare. Cure was never a goal of therapy as it was assumed unattainable. Instead, the goal was to control the disease as much as possible, providing the best quality of life to patients for the longest duration by judicious, intermittent use of the 2 available classes of active chemotherapeutic agents. Also in 1986, clinical studies evaluating HDT with single ASCT (McElwain) and double ASCT (Barlogie) were conducted. In 1996, the first randomized study showed benefits with HDT with ASCT versus standard chemotherapy. Berenson et al described an efficacy of bisphosphonate pamidronate in reducing skeletal events in patients with advanced MM^.^

**Fig. 1:**
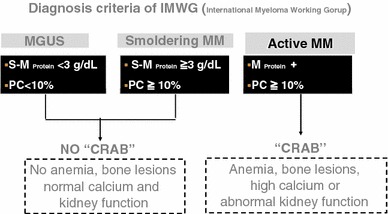
Diagnostic criteria of IMWG. Anemia, bone lesions, high calcium or abnormal kidney function are called “CRAB”. We start any initial treatments at the symptomatic myeloma. MGUS and smoldering myeloma are only careful following

In 1999, thalidomide was introduced and the first non-myeloablative mini-allogeneic transplants were introduced with several novel agents that target the biological pathway of the disease, as well as long-acting Adriamycin^®^ analogues. In the past decade, thalidomide, bortezomib, and lenalidomide have emerged as effective agents for the treatment of myeloma, producing spectacular results in combination with other known agents in terms of response rate, CR rate, progression-free survival (PFS), and, more recently, overall survival. In 2001, a new classification system introduced “CRAB” features of organ damage (Fig. 1) [5]. In 2004, the International Staging System was introduced. The results obtained from new combinations have indeed been remarkable and have created a relatively new philosophy of treating myeloma with a goal of potential cure rather than disease control.

Chemotherapy is indicated for patients with newly diagnosed symptomatic myeloma, although it is generally not recommended for patients with monoclonal gammopathy of undetermined significance (MGUS), smoldering, or asymptomatic myeloma. Age, performance status, and neurologic and co morbid conditions are critical factors in the choice of initial therapy. Melphalan and prednisone combination can no longer be considered as a standard of care in patients who are 65 years of age or older. Our findings suggest that bortezomib plus melphalan-prednisone is the standard front-line treatment for patients with myeloma who are 65 years of age or older and cannot tolerate more aggressive treatment [6].

During the past decades, high-dose therapy with autologous stem-cell transplantation (HDT-SCT) has become the standard treatment option for patients with untreated multiple myeloma (MM) who are younger than 65 years of age; however, HDT-SCT is not usually recommended for older patients and patients with clinically significant co-morbidities.

A recent study has shown that long-term survival improved significantly in younger patients while only limited improvement was achieved in elderly patients. Improved treatment for such older patients ineligible for HDT-SCT was much-awaited.

Should we treat patients with myeloma with multidrug, multitransplant combinations to pursue the goal of potentially curing a subset of patients, recognizing that the balance of adverse events and effect on quality of life will be substantial? Or should we consider myeloma as a chronic incurable disease with a goal of disease control, using the least toxic regimens, emphasizing a balance between efficacy and quality of life, and reserving more aggressive therapy for later lines?

## Induction therapy for newly diagnosed multiple myeloma (NDMM)

**Fig. 2:**
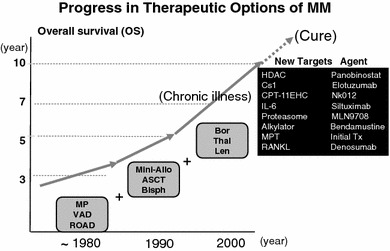
Effect of novel agents on outcome in newly diagnosed myeloma. Overall survivals were elongated by the effect of HDT with ASCT from 1994, longer due to new drugs from 2001. 1970, MP; 1986, HDT with ASCT; 1999–2000, new drugs (bortezomib, lenalidomide, and thalidomide) were epoch making. The CS-1 antibody (elotuzumab) and IL-6 antibody (siltuximab) may be effective with some combinations. Bendamustine, a bifunctional agent, shares properties of alkylating agents and purine analogs. New combination trials of new agents, as shown in right-side may be promising

Effect of novel agents on outcome in NDMM was dramatically improved (Fig. 2) [7]. Using the combination therapies with new drugs, multiple myeloma (MM) is changing from a incurable disease into either a chronic one or a curable disease.

### Bortezomib

Bortezomib IV is an ubiquitin-proteasome inhibitor and indicated for the treatment of MM. Bortezomib is a reversible inhibitor of the chymotrypsin-like activity of the 26S proteasome in mammalian cells. It is cytotoxic to a variety of cancer cell types in vitro and causes suppression in tumor growth in vivo in nonclinical tumor models, including MM. Specifically, bortezomib is effective in MM via its inhibition of nuclear factor-κB activation, its attenuation of interleukin-6-mediated cell growth, a direct apoptotic effect, and possibly antiangiogenic and other effects [8]. Regarding the treatment of patients who are not eligible for transplantation, MPT and MPB have shown significantly better overall survival (OS) benefit than that of MP and are the recommended treatments [6, 9]. The proteasome inhibitor bortezomib has been approved in the USA in 2005 for the treatment of MM patients with a history of at least one prior therapy, based on results from the phase III APEX study which showed superiority of bortezomib over high-dose dexamethasone in patients with relapsed MM [10]. The majority of treatment guidelines currently recommend incorporating HDT/SCT into initial therapy programs for patients who are 65 years of age or younger and to consider such a therapy for patients 60–70 years of age with good performance status and a lack of co morbid illnesses since HDT/SCT provides the highest chance of inducing a complete remission. However, even when patients achieve CR, the vast majority of patients will ultimately relapse. The standard frontline therapy for patients who are 65 years of age or older, and for patients who are not likely to proceed to HDT/SCT, consists of oral MP at doses similar to those used in this study. Combination therapies such as MP (at a dose of 0.25 mg/kg/day) are given orally at doses used for 4 consecutive days every 6 weeks, showed superior survival versus melphalan alone. With MP therapy, an OR rate of approximately 50 %, a CR rate of 2 to 5 % and a median time to response of 3–5 months have been historically reported [4].

### Final results of the phase 3 VISTA trial

Recently 5 year OS follow up data has been published. The data indicates that OS in MPB with 60.1 months follow-up is significantly superior to that of MP. The OS of MP-B and MP were 56.4 months (13.3 months improvement) and 43.1 months respectively. This data is very much remarkable because the OS improvement was 13.3 months although even MPT could improve only 6.6 months in its meta analysis. As a result of this VISTA study, MPB became the standard treatment for untreated transplant in-eligible patients [11].

To evaluate safety, pharmacokinetics (PK) and efficacy of bortezomib combined with melphalan and prednisolone (MPB) therapy, we conducted a phase I/II study for untreated Japanese MM patients who were ineligible for hematopoietic stem cell transplant (HSCT). This was a dose-escalation study designed to determine the recommended dose (RD) of bortezomib in combination with melphalan and prednisolone by evaluation of the maximum tolerated dose based on dose-limiting toxicity (DLT) in the phase I portion, and to investigate the overall response rate (ORR; CR + PR) and safety of MPB therapy in the phase II portion. Particularly, a continuity of treatment cycles was historically compared with a global phase III study (VISTA trial), and the incidence of interstitial lung disease was assessed. This phase I/II study in Japan suggests that the RD of bortezomib in MPB therapy is 1.3 mg/m^2^ and the MPB therapy in newly diagnosed Japanese MM patients ineligible for HSCT is as effective as that shown in VISTA trial. Further investigation is necessary to confirm the appropriate administration schedule of this combination in Japanese patients [12].

**Fig. 3:**
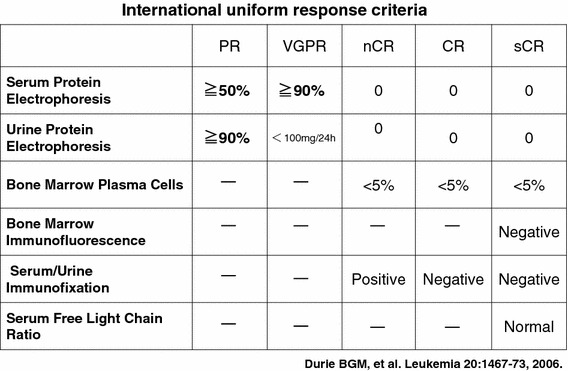
International uniform response criteria. Serum protein electrophoresis, serum/urine immunofixation, and serum free light chain ratio are important

**Fig. 4:**
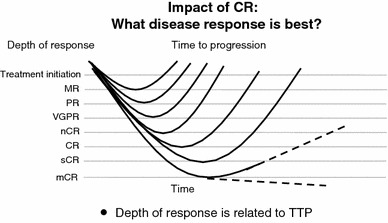
Impact of CR: depth of response is related to TTP. CR is the surrogated marker for the long survival

What should be the goal of treatment in multiple myeloma? If cure is the goal, then CR is the critical first step (Fig. 3) [13]. CR is a treatment goal in many hematological malignancies, eg- AML, ALL and lymphomas. In the past, achievement of CR in MM was rare. New treatments can increase the rate of CR to the similar level with high-dose therapy followed by ASCT (Fig. 4) [14–16]. Also, CR rate in Phase 3 trials in non-transplant patients was: MPB 30 %; MPT 2-16 %; MPR 13 %; MPR-R 18 %, and long term RD 22 %. MM may not be a single disease cytogenetically; achievement of CR seems particularly important in the 15 % of patients with high-risk MM, since survival is similar in patients without high-risk features who have and have not achieved CR [6, 17–20].

### Cyclophosphamide and thalidomide

Cyclophosphamide has been added to thalidomide and dexamethasone (CTD) with excellent response rates among newly diagnosed MM patients who received subsequent SCT, with higher response rates seen after SCT.

The combination in 3-weekly schedules of cyclophosphamide (50 mg PO or 300 mg/m^2^ PO weekly or 150 mg/m^2^ d1–5), thalidomide (200–800 mg daily, increasing doses or intermittent administration 400 mg d1–5 and d14–18) and dexamethasone (40 mg per day for 4 days) (CTD) results in an ORR of around 60 %, a median TTP of 10–12 months and a 2-years PFS of 57 % [21–23].

Comprehensive reviews on the use of thalidomide have been published and include efficacy and safety in relapsed MM. The rationale for using thalidomide was based on its antiangiogenic properties because, in MM, increased microvessel density has been inversely correlated to survival. However, thalidomide has multiple modes of action, including immunomodulatory effects. This initial experience generated a great enthusiasm, and a large number of phase II trials were rapidly conducted. A systematic review of such 42 trials on >1600 patients confirm that the response rate is 29 % with an estimated 1-year overall survival (OS) of 60 %. The well-known teratogenicity of thalidomide is not a major concern in patients with MM because of patients age, but justifies careful informing of patients and programs to avoid drug exposure in women with childbearing potential. The major toxicities of thalidomide are fatigue, somnolence, constipation, and mostly peripheral neuropathy, which are related to the daily dosage and to treatment duration. The overall incidence of peripheral neuropathy is 30 % but may be higher if treatment is prolonged for >1 year. Because this complication may be disabling and sometimes irreversible, patients should decrease the dose or stop the treatment if significant numbness occurs.

After induction treatment, two to four cycles of combination therapies is followed by the maintenance therapy, which is continuous therapy with a single agent, with reasonable balance between maximum benefits and minimum toxicities [24] until the time of disease progression.

## Maintenance therapy for multiple myeloma

**Fig. 5:**
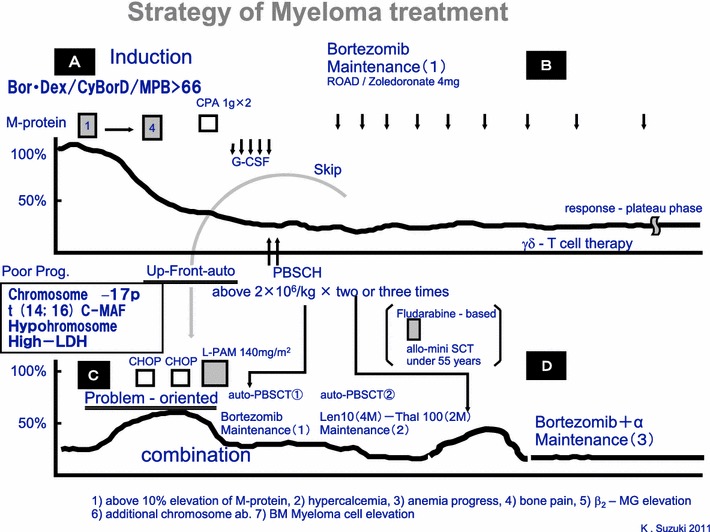
Strategy of myeloma treatment in our institute. We divided in four phases: initial therapy by two to four courses of BorDex/CyBorD/ or MPB >66 years old followed by PBSC-harvest. If the high risk patients, up-front PBSC-transplantation followed by Bor-maintenance. Otherwise, if the standard risks patients, maintenance-therapies may be the B-stages until progress disease. PD are defined as (1) above 10 % elevation of M-protein, (2) hypercalcemia, (3) anemia progress, (4) bone pain, (5) β_2_-MG elevation (6) additional chromosome ab. (7) BM myeloma cell elevation. After PD, problem-oriented PBSCT may be done with second maintenance with Lenalidomide

I prefer disease control as a treatment goal, except in selected high-risk patients in whom an aggressive approach to achieving CR may be the only option to long-term survival (Fig. 5). The disease control approach involves targeting very good partial response (minimal residual disease) rather than CR as a goal by using limited, less intense therapy first and moving to more aggressive approaches as need arises (sequential approach): this allows patients to help determine the timing and number of transplants.

Post-transplant consolidation/maintenance with novel agents can become an important step forward. Thus, it has recently been reported that post-transplant consolidation with thalidomide, lenalidomide or bortezomib increases the CR rate. In this regard, it has been shown that post-ASCT consolidation with VTD can induce long-lasting molecular remission [25, 26]. Thalidomide maintenance prolonged the OS in two transplant series [27].

The response rate to treatment with single-agent thalidomide in patients with relapsed and/or refractory MM is between 30 and 40 % [28].The response rate increases from 50 to 65 % when thalidomide is combined with dexamethasone with or without cytotoxic agents.

The cure-versus-control debate is hot. Indeed, CR is a surrogate marker for improved OS. However, for the majorities of MM patients, the disease control approach (Maintenance therapy) involves targeting very good partial response (VGPR) rather than CR as a goal. This is a pilot study of the prospective, sequential registered trial of the significance of BD maintenance therapy for long-term survival with good QoL.

**Fig. 6:**
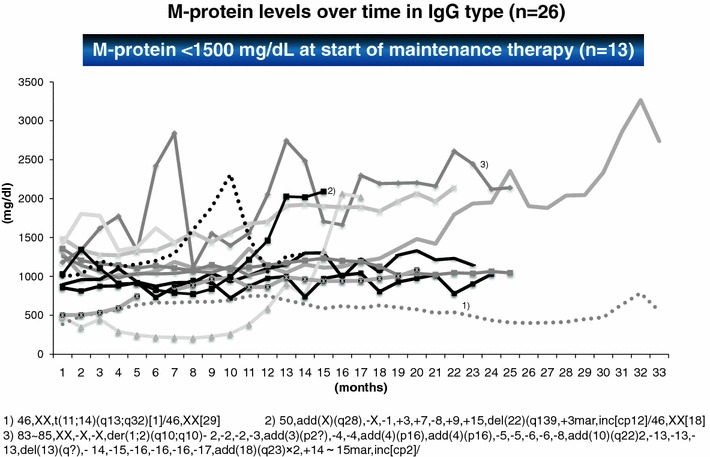
Maintenance therapy with bortezomib for the VGPR IgG-myeloma patients. Monthly administration of bortezomib are effective as the stabilization of M-protein levels over time in IgG type (*n* = 26). It needs 20 months average until PD. However, high risk group are difficult to control in this manner

**Fig. 7:**
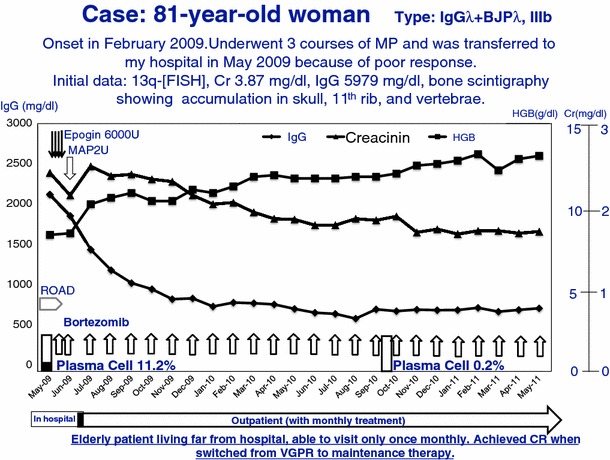
A case of Bor-maintenance therapy. Elderly patient (81 year old female: IgGλ + BJPλ, stage IIIb) living far from hospital, can visit only once monthly. After 14 months therapy, she achieved CR when switched from VGPR to maintenance therapy

From September 2008, we continued exploratory study of effects of bortezomib on the ability of patients with relapsed, refractory multiple myeloma to continue maintenance therapy [29] (Clin. Eth. No: JRC 170). Bortezomib had been associated with fatal lung disorders, with a high number of reported cases in Japan. Post-marketing surveillance, however, showed a low incidence of 3.6 %. Peripheral neuropathy (20–30 %) is a major concern. Informed consent was obtained from 43 patients with a mean prior treatment (e.g., VAD, ROAD, ASCT) history of 23 months, PS ≤2, and no significant organ lesions. Efficacy of bortezomib as maintenance therapy in patients achieving VGPR/PR with remission induction therapy has not been investigated. This study of bortezomib maintenance therapy in patients achieving VGPR/PR with bortezomib is therefore investigating the effects of treatment on patients ability to continue maintenance therapy and adverse drug reaction incidence. There were 11 cases of karyotypic abnormalities (35 %) with 8 cases of complex abnormalities. Patients received dexamethasone (20 mg/body) daily for 2 days every 2 or 4 weeks with bortezomib, 1.3 mg/m^2^ div. Time-to-progression (TTP) was the primary efficacy endpoint (Fig. 6) [29]. The adverse reactions of BD maintenance include asthenia conditions, peripheral neuropathy, thrombocytopenia were all G-1 and well tolerated. Long-term survival with good QoL is the most important goal for the elderly/low genetic risk MM patients. BD maintenance is good available for this group (24/43 cases) over 20 months (Fig. 7), especially in the cases of total delivery dose over 40 mg. However, the other group of patients (8/33 cases) in rapidly relapsing with complex karyotypic abnormalities may need the strong combination chemotherapy.

Recently, lenalidomide maintenance therapy improved median progression-free survival (41 vs. 23 months with placebo; hazard ratio, 0.50; *P* < 0.001) [30].

## Therapy for relapsed or refractory multiple myeloma (RRMM)

Progressive disease is defined as follows: (1) Above 25 % elevation of M-protein, (2) hypercalcemia: corrected serum calcium >11.5 mg/dL, (3) the absolute increase of free light chain (FLC) must be >10 mg/dL, (4) definite development of new bone lesions or soft tissue plasmacytomas, (5) decrease in hemoglobin of >2 g/dL, (6) rise in serum creatinine by 2 mg/dL or more, (7) increase of BM myeloma cell above 10 %.

### Analysis of second primary malignancies (SPM)

Another important issue in MM is risk of developing SPMs due to living longer from diagnosis. Population studies show MM patients have increased risk of specific SPMs following initial diagnosis, notably acute myeloid leukemia (AML). Some MM therapeutic agents are particularly associated with elevated risk of SPMs. Melphalan is associated with increased risk of secondary acute leukemia. There were imbalances in SPM incidence, including myeloid and lymphoid leukemias, with post-transplant lenalidomide maintenance therapy and with MP-lenalidomide. Persistent significant OS benefit with VMP versus MP; 13.3-months increase in median, and MPT versus MP increase 6.6 months [9].

Secondary malignancies and lenalidomide: by summarizing the data to-date, the incidence of all/invasive SPM is significantly increased in Lenalidomide arms, driven by hematologic SPM (*P* < 0.001). B-ALL, Hodgkin lymphoma is reported in post high-dose melphalan and ASCT setting. Sensitivity analysis (including SPM as an event) demonstrates negligible PFS differences. The overall benefit–risk profile of lenalidomide in NDMM remains positive [31, 32]. Risk Factors for Secondary Malignancies Treatment with lenalidomide may be treatment duration >24 months, male, age >55 years, ISS stage III, previous DCEP (role of concomittant or previous exposure to alkylators?) induction by univariate and multivariate analysis in IFM 2005.

In Japanese SPM Report by JRCMC, retrospective analysis for 325 MM patients from 1998 to 2010 (13 years) showed t-MDS/AML developed 17 (5.2 %) patients. Median time to onset: 52 months in t-AML and months in t-MDS. All the patients with t-AML died in a short time, suspected to be treated with Melphalan, and no patients had been given Lenalidomide. We have to select chemo regimen taking into account the risk of t-MDS/AML [33].

### Renal dysfunction in multiple myeloma

**Fig. 8:**
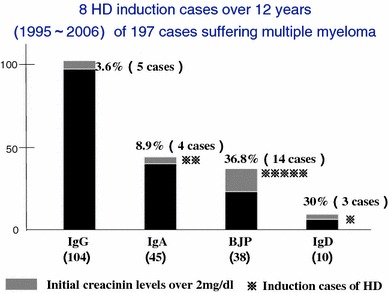
HD induction cases suffering MM. Initial creatinine levels over 2 mg/dL were 10–20 %, mainly in BJP and IgD type. HD induction was also frequent in these populations

Timing of treatment initiation in multiple myeloma is depending on existence of organ dysfunction. Usually when any symptom such as bone symptoms, renal dysfunction, anemia, or hypercalcemia is observed, it is diagnosed as symptomatic multiple myeloma and treatment should be started. Renal dysfunction in multiple myeloma is one of the complications that require the most careful attention and occurs via various mechanisms. Of these, the most frequent case is cast nephropathy, also known as myeloma kidney, in which excessive light chains of M protein (BJP) secreted by proliferated plasma cells form cast by depositing themselves in renal tubules. In addition, hypercalcemia associated with osteolysis by myeloma cells, deposition of amyloid in glomeruli, hyperviscosity syndrome, hyperphosphatemia, renal infiltration of myeloma cells are also the causes of renal dysfunction. Other than those, care must be given to recurring urinary tract infection, drugs, dehydration that may act as exacerbation factor. According to the statistics of Japanese Society of Myeloma [34], approximately 15 % of newly diagnosed multiple myeloma patients have complication of renal dysfunction and the rate increases as the disease progresses. Bence Jones protein (BJP) type and IgD type of myeloma that excrete high amount of Bence Jones protein into urine show high frequency of renal dysfunction. In 197 patients diagnosed as multiple myeloma during 12 years (1995–2006) in our facility, 3.6 % of IgG type and 8.9 % of IgA type showed higher than 2 mg/dL of creatinine on the first visit, were whereas BJP type accounted for 36.8 % (Fig. 8). Because renal dysfunction becomes irreversible if timing of treatment is missed, immediate treatment is necessary. It is reported that renal dysfunction remains reversible when serum creatinine is below 4 mg/dL, Ca is below 11.5 mg/dL and urine protein is 1 g/day or lower [35]. Although these are the data before introduction of novel agents, in the 423 patients with newly diagnosed multiple myeloma, patients with renal dysfunction (22 %) showed significantly shorter survival time compared to the patients with normal renal function (8.6 vs. 34.5 months). In addition, Blade et al. reported that in the same patients with reduced renal function, those who recovered their renal function by subsequent chemotherapy showed significantly extended survival time compared to those without recovery of renal function (28.3 vs. 3.8 months). Therefore, although renal dysfunction in multiple myeloma is a poor prognostic factor, good prognosis can be expected if the treatment restores renal function. For this, it is important to restore renal function by implementing effective treatment in patients with renal dysfunction before it becomes irreversible and requires hemodialysis. In the multiple myeloma patents in our facility mentioned above, hemodialysis was introduced to eight out of 197 cases.

### Improvement of renal function and treatment strategy for multiple myeloma

**Fig. 9:**
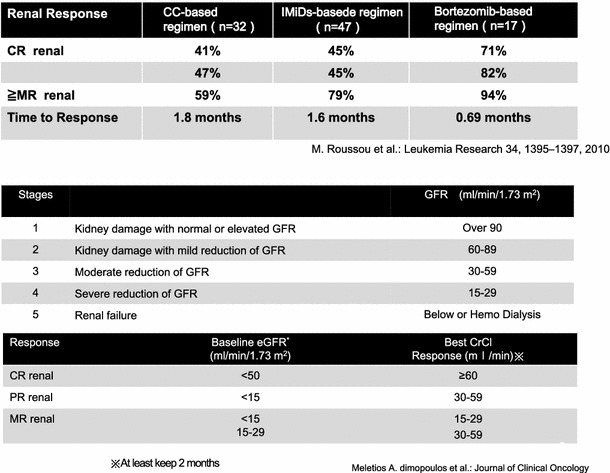
Complete response (CR) renal. CR may be attained by bortezomib-based regimen not only the high levels percentage but also time to response. 5-stage is divided as the figure

Improvement of the primary disease is the basic remedy of renal dysfunction that complicates with multiple myeloma. Since 2005, treatment strategy for multiple myeloma has significantly changed due to the successive introduction of novel agents. The three drugs including a proteasome inhibitor bortezomib, and two immunomodulatory drugs (IMiDs), lenalidomide and thalidomide, are referred to as novel agents, and each drug has characteristic profiles of efficacy and safety. While all those agents can be expected to restore renal function due to improvement of the primary disease, bortezomib, with strong antitumor effect, is reported to rapidly improve renal function (Fig. 9). Roussou et al. retrospectively compared improvement of renal function among traditional chemotherapy group, IMiDs (lenalidomide or thalidomide)-based treatment group, and bortezomib-based treatment group with 96 cases of newly diagnosed multiple myeloma. It showed that the best and the most rapid improvement of renal function were observed in the bortezomib-based treatment group. Renal response rate (minor response and better) based on creatine clearance improvement and time to response as 59 % and 1.8 months in chemotherapy group, 79 % and 1.6 months in IMiDs-based group, and 94 % and 0.69 month in bortezomib-based group, respectively [36]. In addition, some cases with withdrawal from dialysis are also reported. Thus, administration of bortezomib should be considered in patients with acute or severe renal dysfunction if it is possible.

### Lenalidomide

Lenalidomide is an anti-myeloma drug possessing dual functions of antitumor effect and immunomodulating activity. Because lenalidomide is urinary excreted, its blood concentration increases in patients with renal dysfunction which leads to high incidence risk of adverse reactions [37]. However, lenalidomide itself has no renal toxicity and clinical studies showed improvement of renal function in the patients treated with lenalidomide. Lenalidomide can be administrated by proper adjustment of its dose corresponding to renal function according to the package description [38]. In fact, it is reported that adjusted dosing of lenalidomide to patients with renal dysfunction resulted with similar anti-myeloma efficacy to those with normal renal function [39, 40], and recovery of renal function was also observed [41]. Similar to bortezomib, cases that withdrew from dialysis are reported [42]. Stratified analysis of lenalidomide/dexamethasone therapy by age showed similar efficacy and tolerability in elderly (over 65 years of age) to those of youth [43]. Hence this therapy is considered to be useful especially for elderly patients with renal dysfunction if the dose is properly adjusted corresponding to the renal function. Thalidomide does not require dose control depending on renal dysfunction, but it has not been reported in large studies that thalidomide is effective on the improvement of renal function. In any case, early diagnosis and timing of initiation of treatment are important. In addition, full understanding of efficacy and safety profiles of novel agents and using them in combination with existing drugs appropriate for individual patients are the basis of treatment strategy.

## Diagnosis of AL amyloidosis and renal dysfunction

**Fig. 10:**
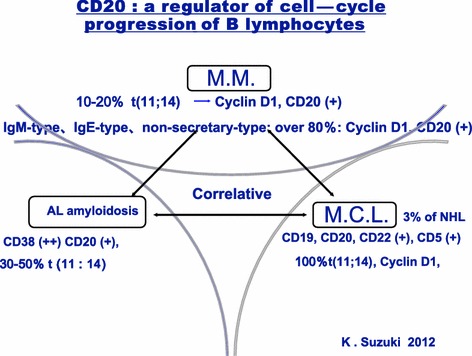
Correlation of pathogenesis between MM, AL amyloidosis and Mantle cell lymphoma by the up-regulated cyclin D1 function. Mantle cell lymphoma is high tumor growth with 100 % t (11:14), MM have 10–20 % t (11:14) with moderate growth and secretary Ig functions. Some strange and rear MM patients (i.e. IgM-type, IgE-type, non-secretary-type) showed translocation 11:14 over 80 %. Otherwise, AL amyloidosis showed 30–50 % t (11:14). There may be the differences of break points on the translocation foci

AL amyloidosis is a disease with poor progression in which deposition of amyloid causes multiple organ failure. Amyloid consists of immunoglobulin light chains secreted from monoclonal proliferated plasma cells. Its relative disease MM is often complicated with AL amyloidosis. In spite of the fact that it has the same chromosome translocation such as t (11:14) to MM, it shows different pathological condition (Fig. 10). This may be due to slight difference of translocation breakpoint between AL amyloidosis and MM. However, the disease mechanism remains unknown.

**Fig. 11:**
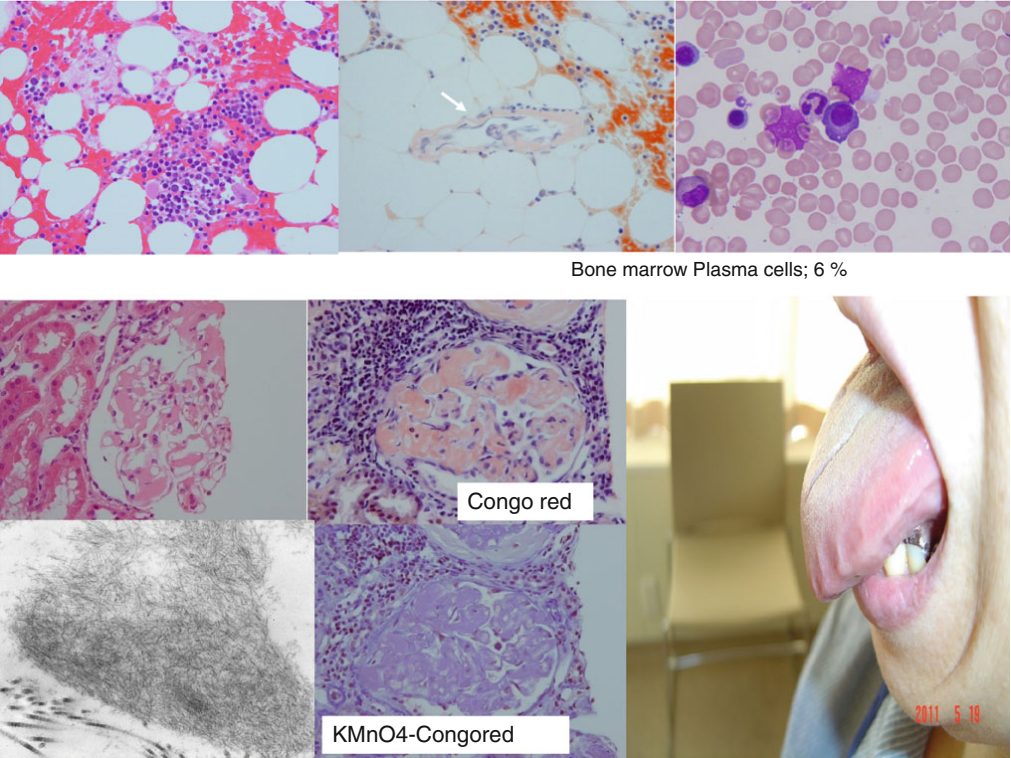
Histology of bone marrow and kidney. Tubercle in margin of tongue is important finding for diagnosis. The amyloidogenic plasma cell clone is mature type mainly CD19 negative clone. We can see amyloid deposition in blood vessels of bone marrow in some cases. Congo-red staining and amyloid fibrils by EM is important by the low detection with light chain staining

It is classified to cardiac, renal, gastrointestinal, and pulmonary amyloidosis depending on the main organ with amyloid deposition. The symptoms vary and the most common cause of death is cardiac failure. The diagnosis is based on confirmation of amyloid deposition in the involved organs. When AL amyloidosis is suspected in patients with clinical findings such as general malaise, edema, heart failure, tubercle in margin of tongue, and skin nodule with stigma, biopsy of organs should be first conducted to confirm deposit of amyloid (Fig. 11). Amyloid is positive with Congo red stain and has positive signal under polarized light with the polarizing filters. AL amyloidosis is definitely diagnosed by confirming monoclonal proliferation of plasma cells through identification of M protein and/or staining pattern of cell surface antigens in addition to deposition of amyloid. Low detection sensitivity of M protein even in immunofixation in AL amyloidosis has been a problem so far. However, the free light chain (FLC) assay that has listed itself in insurance coverage in 2011 in Japan, allows over 90 % detection and is reported to be effective in diagnosis. Amyloid deposits are predominantly composed of amyloid fibrils which are very stable structures with a common cross core fold. Deposits are always rich in proteoglycans and glycosaminoglycans, some of which are tightly associated with the fibrils and further stabilize them against proteolytic degradation by phagocytes and affinities of selective organ deposition..

Renal dysfunction in AL amyloidosis is frequently caused by glomerular injury due to deposit of amyloid and observes high albuminuria and nephrotic syndrome. Its progression leads to kidney failure, and in many cases requires dialysis.

## Therapy of AL amyloidosis

**Fig. 12:**
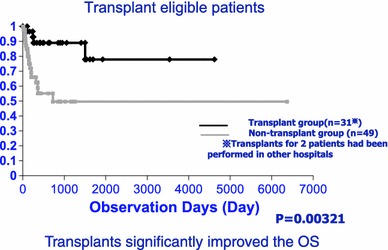
Autologous stem cell transplantation (ASCT) for AL amyloidosis. ASCT in the early stage of AL amyloidosis is effective for the OS and good QOL. In our experiences, group of ASCT showed good OS compared with the others (*P* = 0.00321)

The target of chemotherapies is the amyloidogenic clonal plasma cells in the bone marrow. Complete remission is the normalized kappa/lambda ratio of serum FLC, the surrogate markers. Similar to MM, the recovery of function in the damaged organ requires the improvement of primary disease. However, the recovery from renal dysfunction with amyloid deposits requires a longer complete remission period. High-dose chemotherapy followed by autologous peripheral blood stem cells (ASCT) is effective in treating AL amyloidosis (Fig. 12).

**Fig. 13:**
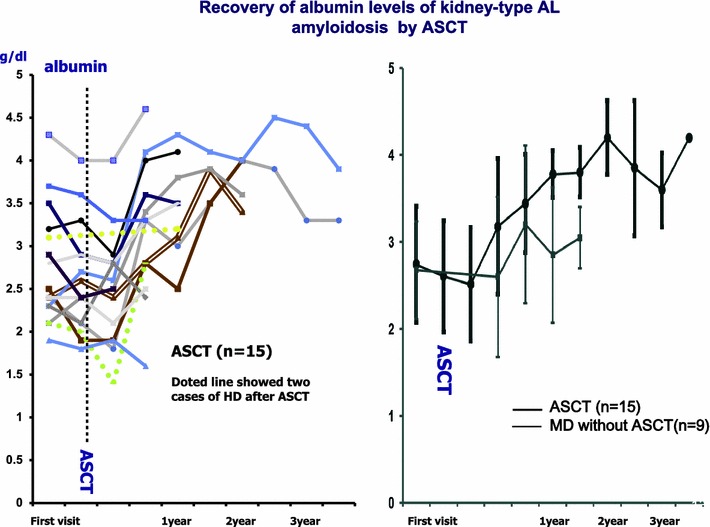
Effect of ASCT for renal type of AL amyloidosis. Early recoveries of the albumin concentration occurred by ASCT in the early stage

The response criteria are roughly classified into hematological response comprised of elimination of M protein, etc. and organ response. In case of renal dysfunction, it is judged by decrease of albumin. The four-year survival rate in transplantation group and non-transplantation group is 71 and 41 %, respectively, showing higher survival rate in transplantation group [44], and in the patients who survive over 1 year and obtain complete remission after ASCT, over 10 years of prognosis can be expected [45]. In our faculty, we conducted high dose chemotherapy with ASCT during 2005–2010 in 15 patients with renal amyloidosis who were 65 years old or younger and had good PS, and every case showed good results (Fig. 13). Poor prognostic factors in high-dose chemotherapy are poor PS, symptomatic cardiac failure, organ failure in more than two organs (heart and kidney), and old age (over 65 years of age), and these cases are non-transplant candidates [46]. MD (melphalan and dexamethasone), thalidomide (Thal/Dex), cyclophosphamide-thalidomide (CTD), and the combinations of MM therapy are the first option for the transplant ineligible. In MD therapy, approximately 60–70 % of hematological improvement and approximately 50 % of improved organ were observed [47]. In overseas, clinical studies are conducted on novel agents (lenalidomide, thalidomide, and bortezomib) of myeloma in combination with melphalan, dexamethasone and cyclophosphamide against AL amyloidosis. Of these, bortezomib is considered most promising because improvement of organs can be expected in addition to its rapid hematological improvement with high rate. On the other hand, peripheral neuropathy and cardiotoxicity were reported as major adverse events of bortezomib, patients have to be carefully observed with these complications. Lenalidomide shows poor tolerability in AL amyloidosis patients at 25 mg/day which is a standard dose in multiple myeloma, and its MTD is 15 mg/day in AL amyloidosis. Around 50–70 % of hematological improvement and around 20–50 % of improvement in organs was reported in lenalidomide therapy of AL amyloidosis [48, 49]. Appropriate use of lenalidomide depending on the state of patients should be considered because it has a different profile of adverse events from bortezomib. Because thalidomide and lenalidomide were reported to worsen renal function in patients with renal amyloidosis, careful monitoring should be given when used in such patients. Transplantation of the involved organs is also an option in the overseas.

## Conclusion

As mentioned above, the therapy and treatment strategy of MM and AL amyloidosis have largely changed in these recent years. At same time, it is becoming more important to control the disease in a long-term fashion, maintaining QoL of patient because it is still difficult to cure the disease. The increase in the number of treatment options means that personalized medicine which selects a treatment corresponding to the systemic condition of the patient, and the purpose of the treatment will be more important. It is important to treat MM as chronic disease by taking into full consideration efficacy and safety of novel drugs and by effectively combining them with existing drugs. Also we should consider how we could help patients through the treatment to live long actively in the society.

MM and AL amyloidosis are caused by functional abnormality of monoclonal plasma cells, and high-dose chemotherapy supported with autologous peripheral blood stem cells is effective to these diseases. However, they are still difficult to be cured and require long-term disease control. In recent years, introduction of novel agents has changed their treatment strategies.

Better understanding of the biology of the amyloidogenic plasma cell clone and the molecular mechanisms underlying the light chain misfolding, tissue targeting and toxicity will define disease-related prognostic criteria. Risk-adapted therapeutic strategies may be required.

However, it is important to take these diseases as chronic diseases. For this purpose, early diagnosis and timing of initiation of treatments is important. Moreover, understanding of characteristics of novel agents and using them in combination with existing drugs appropriately for individual patient is critical. In addition, collaboration with renal medicine is essential to avoid introduction of dialysis. Also we should consider how we could help patients by treatment to live long actively in the society.
